# Retinal Blood Vessel Formation in the Macula Following Intravitreal Ranibizumab Injection for Aggressive Retinopathy of Prematurity

**DOI:** 10.7759/cureus.60005

**Published:** 2024-05-09

**Authors:** Hiroshi Kubota, Yoko Fukushima, Andira Bulan Nandinanti, Takao Endo, Kohji Nishida

**Affiliations:** 1 Ophthalmology, Higashiosaka City Medical Center, Higashiosaka, JPN; 2 Ophthalmology, Osaka University Graduate School of Medicine, Suita, JPN; 3 Ophthalmology, Osaka Women's and Children's Hospital, Izumi, JPN

**Keywords:** retinal blood vessels, preterm born infant, angiogenesis, anti-vascular endothelial growth factor treatment, retinopathy of prematurity

## Abstract

Retinopathy of prematurity (ROP) is a leading cause of childhood blindness. Recently, anti-vascular endothelial growth factor (VEGF) drugs have been widely used for ROP to inhibit abnormal retinal angiogenesis. However, there is a concern that such drugs potentially also affect normal retinal vascular development. We report a case of blood vessel growth across the macula after anti-VEGF treatment for zone I aggressive ROP. A 25-week-old female infant was administered 0.2 mg of ranibizumab for bilateral aggressive ROP in both eyes at 33 weeks of postmenstrual age. Under normal development, retinal blood vessels do not grow into the center of the future macular region. After five weeks, however, a horizontal blood vessel sprouted from the optic disc and extended across the macula in the right eye. The blood vessel ran straight to the vascular-avascular juncture by 41 weeks of postmenstrual age during the follow-up period. While the focus has been on arresting retinal vascular development through VEGF inhibition, anti-VEGF treatment may induce vascular abnormalities in patients with severe ROP. Infants with retinal vascular abnormalities should be carefully monitored for their visual prognosis.

## Introduction

Retinal blood vessels grow toward the avascular retina in physiological angiogenesis during the developmental stage [1]. However, in pathological angiogenesis, disorganized blood vessels grow into the vitreous, which is observed in patients with retinopathy of prematurity (ROP). These abnormal vessels can lead to retinal detachment and hemorrhage, resulting in blindness [2]. In recent years, anti-vascular endothelial growth factor (VEGF) drugs have been widely used to suppress these abnormal vessels in ROP [3]. On the basis of several clinical trials demonstrating that anti-VEGF therapy is comparable to laser photocoagulation, it has become increasingly common to treat ROP patients with such drugs as an initial treatment [4].

In proliferative diabetic retinopathy, a common retinal disease in adults characterized by neovascularization, anti-VEGF drugs rapidly regress abnormal vessels but do not redirect them toward the avascular retina [5]. By contrast, in ROP, anti-VEGF drugs not only regress abnormal vessels but also subsequently induce vascular regrowth toward the ischemic retina [6]. While anti-VEGF therapy offers advantages for retinal vascular development, it presents new challenges in terms of vascular extension delay [2,7]. Ultimately, retinal vascularization remains incomplete, leading to a condition termed persistent avascular retina (PAR) [6]. Studies have reported that the incidence of eyes with PAR after anti-VEGF therapy ranges from approximately 22% to 38% [8-10]. Additionally, ROP infants treated with anti-VEGF drugs exhibit vascular changes such as vascular tortuosity, circumferential interconnecting vessels, and non-bifurcation vessel branching in the peripheral retina [6,11].

Therefore, it is possible that anti-VEGF drugs may influence vascular pathfinding and the vascular network in the peripheral retina. By contrast, there have been limited reports on vascular changes in the posterior pole retina. In this study, we present a case of newly formed blood vessels running across the macula after anti-VEGF treatment in a patient with aggressive ROP (A-ROP) in zone I.

## Case presentation

A 25-week-old female infant weighed 480 grams at birth, with Apgar scores of two at one minute and five at five minutes, respectively. At 14 days after birth, the patient required a higher level of care and was transferred to the Neonatal Intensive Care Unit at our hospital, Osaka Women’s and Children’s Hospital, located in Osaka, Japan. The patient had several risk factors for ROP early in life. She received the following treatments: three doses of surfactant for respiratory distress syndrome; two catecholamines (dopamine and dobutamine), diuretics, vasopressin, and corticosteroid for circulatory dysfunctions; intubation and mechanical ventilation targeting oxygen saturation at 95% for a total of 246 days; vancomycin for methicillin-resistant *Staphylococcus aureus* sepsis for six weeks; one course of ibuprofen for patent ductus arteriosus; 12 blood transfusions; 10 platelet transfusions; and insulin for hyperglycemia for six days. Additionally, she was diagnosed with grade I intraventricular hemorrhage and pleural effusion due to lymphangiectasis. Her pleural effusion was refractory without responding to hydrocortisone, prednisolone, octreotide, or sirolimus. She exhibited no major congenital anomalies or dysmorphic features.

**Figure 1:**
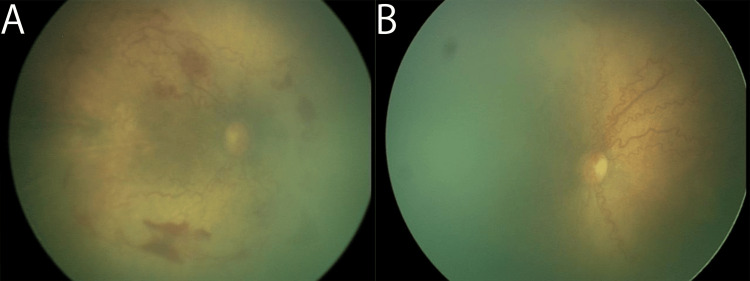
Retinal images of the right (A) and left eyes (B) at the time of treatment Note the poorly vascularized areas in both eyes. Images here and for all other figures were obtained with a RetCam Shuttle (Clarity Medical Systems, Pleasanton, California, USA).

The initial retinal examination was scheduled at a postmenstrual age (PMA) of 30 weeks, but this was postponed according to the decision of neonatologists because the patient was severely ill. The patient exhibited prominent dilated and tortuous vessels, with circumferential shunting and retinal hemorrhages in the retinas of both eyes at the initial screening at a PMA of 33 weeks (Figure 1).

**Figure 2:**
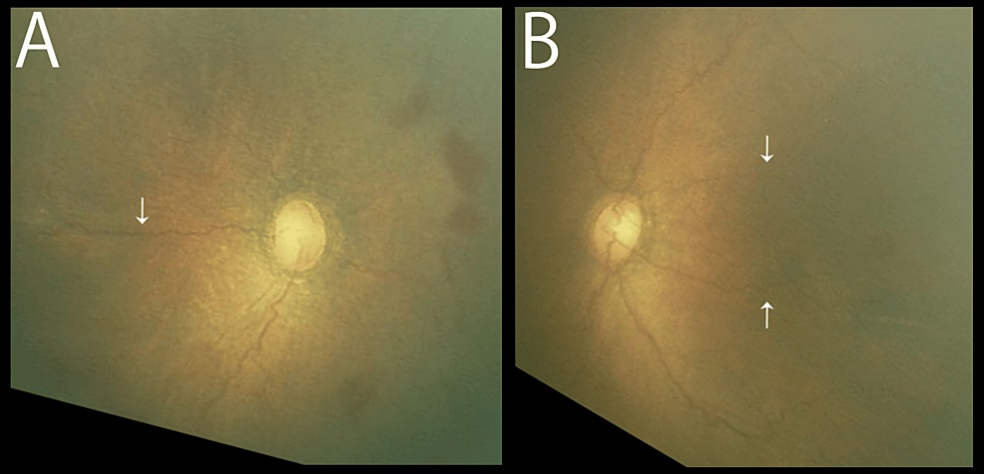
Retinal images of both eyes five weeks after the initial intravitreal ranibizumab (postmenstrual age of 38 weeks) The white arrow indicates a horizontal blood vessel across the macula of the right eye (A) and a horizontal vessel avoiding encroachment into the macula of the left eye (B).

The patient was diagnosed with A-ROP and intravitreally administered 0.2 mg of ranibizumab in both eyes on the same day. In response to anti-VEGF therapy, the tortuous and dilation of vessels rapidly regressed. After five weeks, we observed a horizontal blood vessel that sprouted from the optic disc and extended across the macula in the right eye of the patient and a horizontal vessel from the optic disc that passed sparing the center of the macula in the left eye (Figure 2).

**Figure 3:**
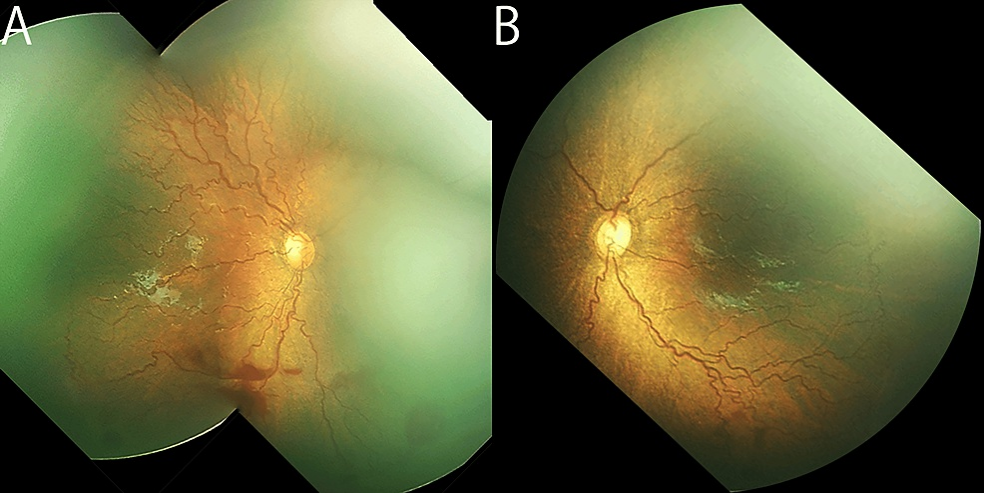
Retinal images show reactivated retinopathy of prematurity eight weeks after the initial intravitreal ranibizumab (postmenstrual age of 41 weeks) Note severe plus disease and ill-defined flat neovascularization in both eyes (A, right eye; B, left eye).

Eight weeks after the first intravitreal ranibizumab (IVR), the macula-crossing vessel was clearly recognized, with an increased diameter and meandering. All other vessels also became tortuous, and flat neovascularization occurred in both eyes (Figure 3).

We considered a second ranibizumab injection for the reactivation (reactivated stage 3 in posterior zone II with plus disease). Although the second IVR-reduced extraretinal proliferation, persistent tortuous vessels remained, and the vascular elongation was suspended. Patient mortality occurred at a PMA age of 66 weeks (six months after the second IVR) due to multi-organ failure. Given that the patient would not have tolerated an examination by fluorescein angiography, we could not conduct this exam throughout the observation period. Chromosome karyotyping test and whole genome sequencing of the patient were completed postmortem. Neither showed any positive results.

## Discussion

Under normal development, retinal blood vessels do not grow into the center of the future macular region, leading to the foveal avascular zone [12]. However, in this infant exhibiting A-ROP, one blood vessel that had terminated between the optic disc and the macula subsequently grew across the macula after IVR. One possible reason is the disruption of the VEGF concentration gradients. During the developmental stage, the VEGF secreted by hypoxic cells diffuses and forms well-defined spatial concentration gradients in the mouse retina [1]. The VEGF gradients facilitate vascular endothelial cell migration toward the peripheral retina [1]. It remains unclear whether the recovery of VEGF levels and concentration gradients after sudden VEGF suppression during retinal angiogenesis is equivalent to those in a healthy condition. Given the efficacy of very low-dose bevacizumab for ROP [13], even if the reactivation rate increases, it would be desirable to use a lower dose than approved or to shift the timing slightly later. To prevent drug-induced avascular abnormalities, it may be necessary to determine the appropriate dosage and timing of anti-VEGF drugs. Since missing the timing of treatment is unacceptable, it may be very useful in the future if intravitreal VEGF levels could be monitored, for example, with tear fluid [14], and the amount and timing could be set.

Among several other possibilities, one possible explanation for the vascular change is foveal hypoplasia. The normal fovea features outward displacement of the plexiform layers in which astrocytes are located. However, the astrocytes are present in the hypoplastic fovea and provide a scaffold that facilitates endothelial cell migration. Consequently, blood vessels may be allowed to cross the fovea. Recent studies have reported that this anomaly could be caused by genetic variants [12,15,16]. In the patient, the whole genome sequencing analysis did not detect any variants associated with foveal hypoplasia. The other possibility is a vascular malformation called congenital retinal macrovessel. The single macrovessel crosses the macula and horizontal raphe, accompanied by surrounding dilated capillaries. The changes in capillaries were not observed in the patient on ophthalmoscope examinations [17]. These congenital anomalies, as well as environmental factors, may be possible. In terms of extrinsic rather than intrinsic causes, unstable systemic hemodynamics after repeated blood transfusions may affect angiogenic factors, such as the concentration of expression of VEGF [18]. However, that would not be directly responsible for the abnormality because all blood transfusions were performed before the first retinal screening.

We could not determine the cause of the vascular abnormality in the eyes because we were unable to perform fluorescein angiography or optical coherence tomography examinations. If the patient had congenital anomalies or foveal hypoplasia, the vessel would have already formed across the macula. The fact that a macula-crossing vessel was newly formed after IVR may indicate VEGF concentration gradient disruption. After anti-VEGF therapy, vascular changes could occur in the posterior pole, as well as peripheral vascular abnormalities such as PAR. Indeed, Rasheed et al. observed capillaries intruding into the macula after IVR in patients exhibiting severe ROP [19]. Further studies are needed to understand the incidence of these vascular abnormalities and their impact on visual prognosis in ROP patients treated with anti-VEGF drugs.

## Conclusions

ROP management is crucial for preventing visual impairment in premature infants. Recent trials have demonstrated that anti-VEGF therapy is comparable to laser photocoagulation. This evidence would promote the application of anti-VEGF therapy because of the associated advantages: ease of use, rapid response, significant reduction of high myopia, and better visual fields. Since the emergence of off-label anti-VEGF drug use for ROP, ophthalmologists have discussed concerns regarding arrested retinal vascular development after VEGF inhibition. In addition to arrested development, anti-VEGF treatment may induce vascular abnormalities in patients with severe ROP. Therefore, infants treated with anti-VEGF drugs should be carefully monitored for vascular changes in the entire retina and their visual prognosis, and parents should be alerted about such potential side effects before consenting to the IVR.
